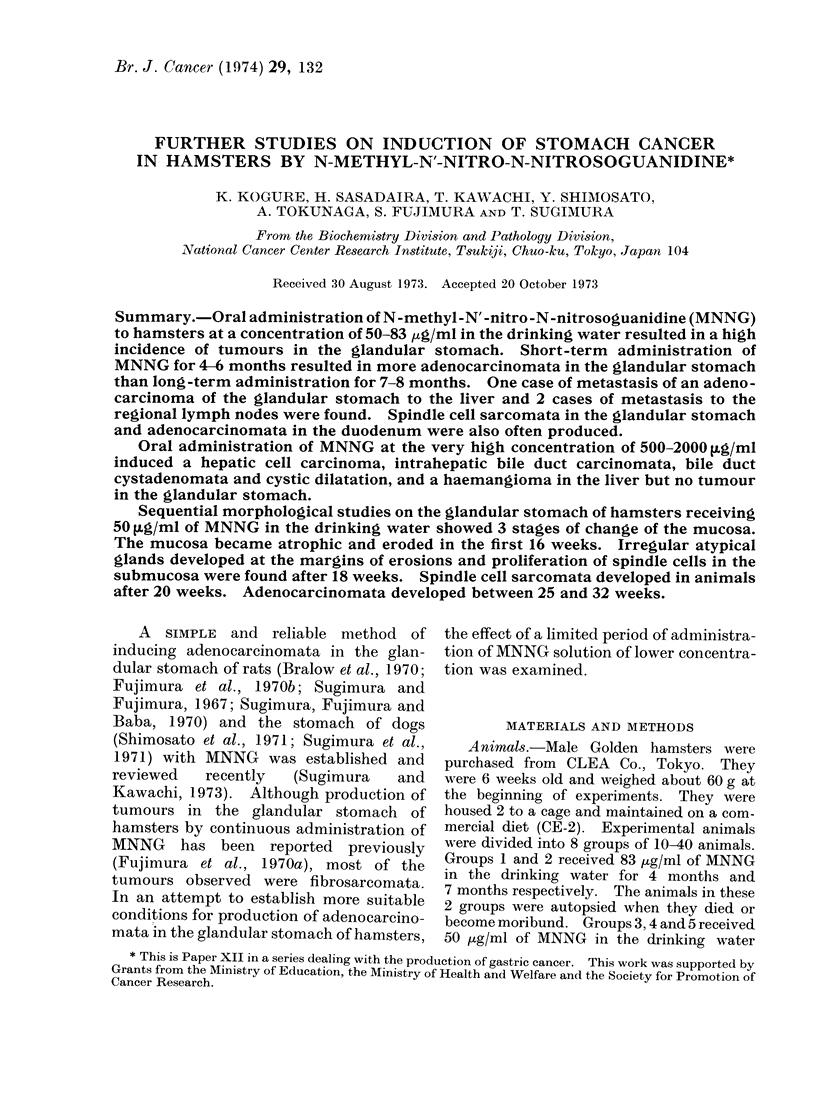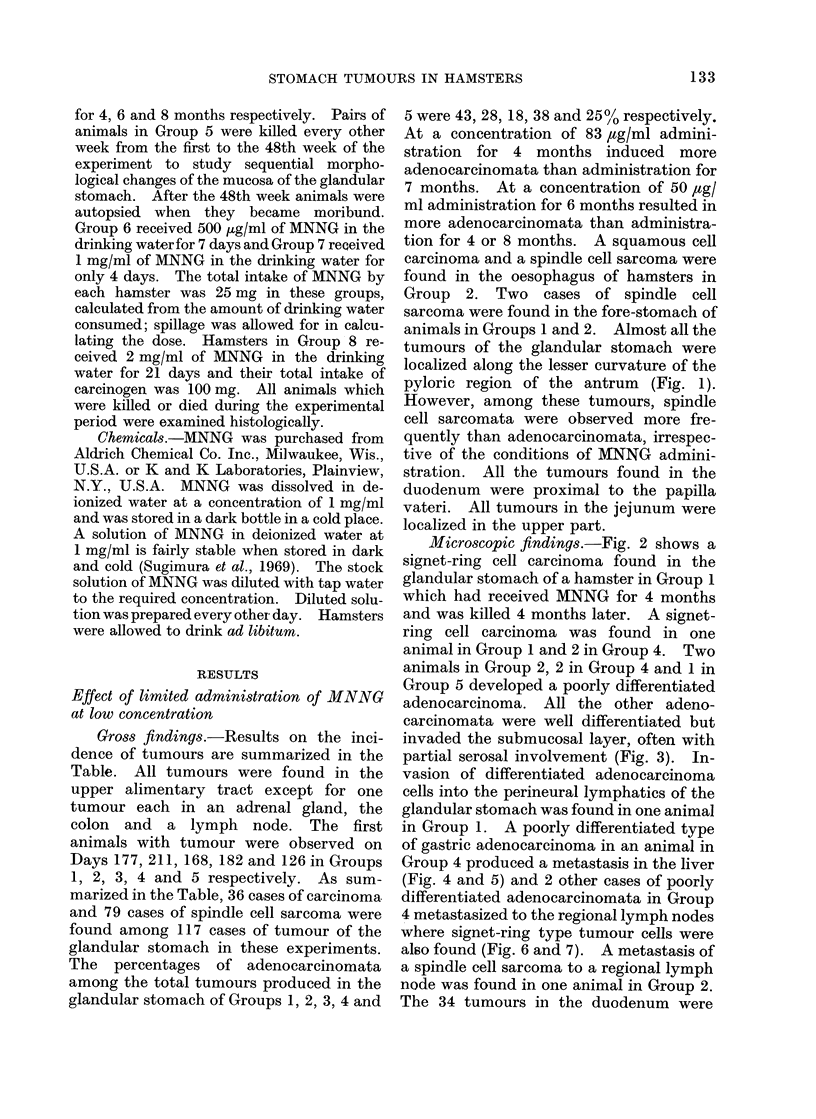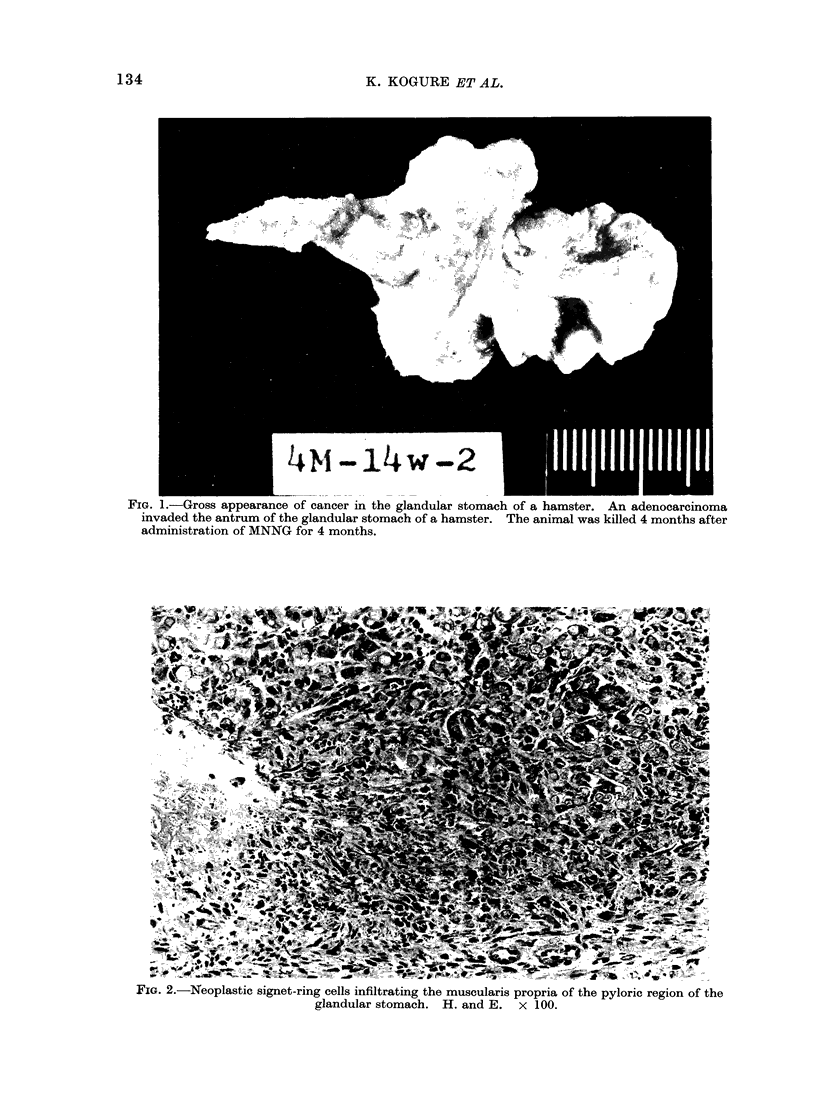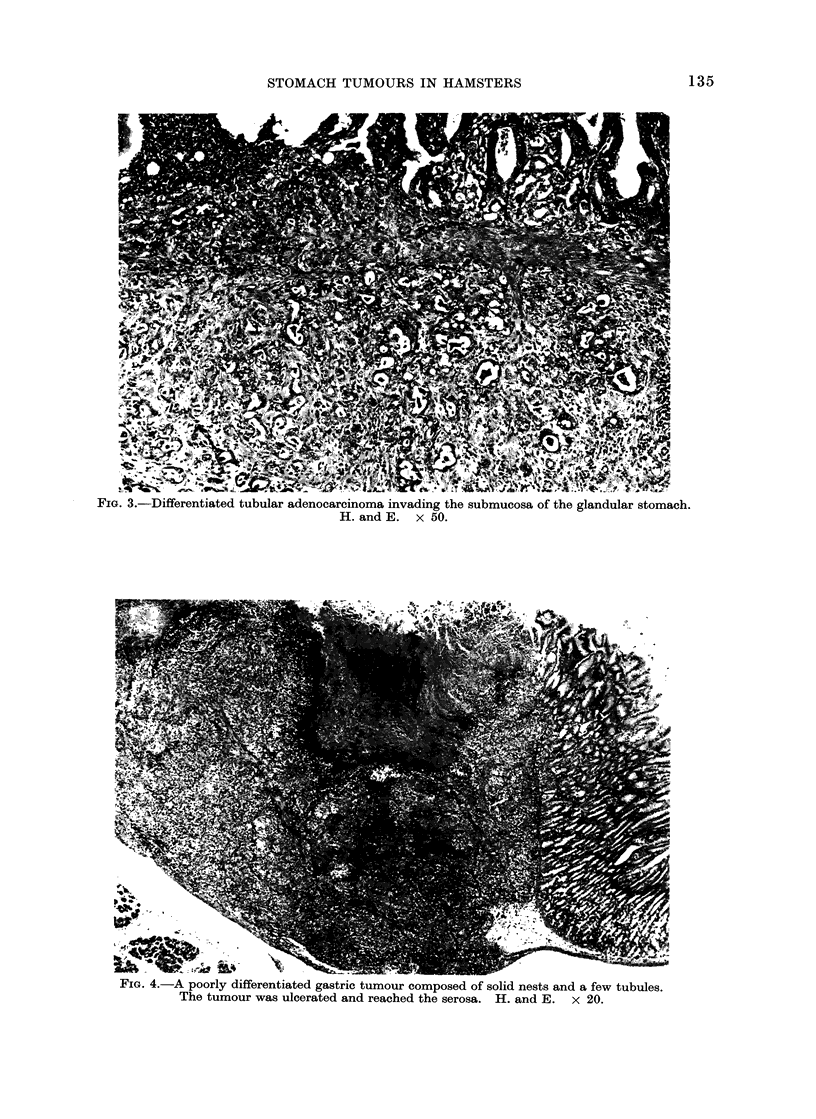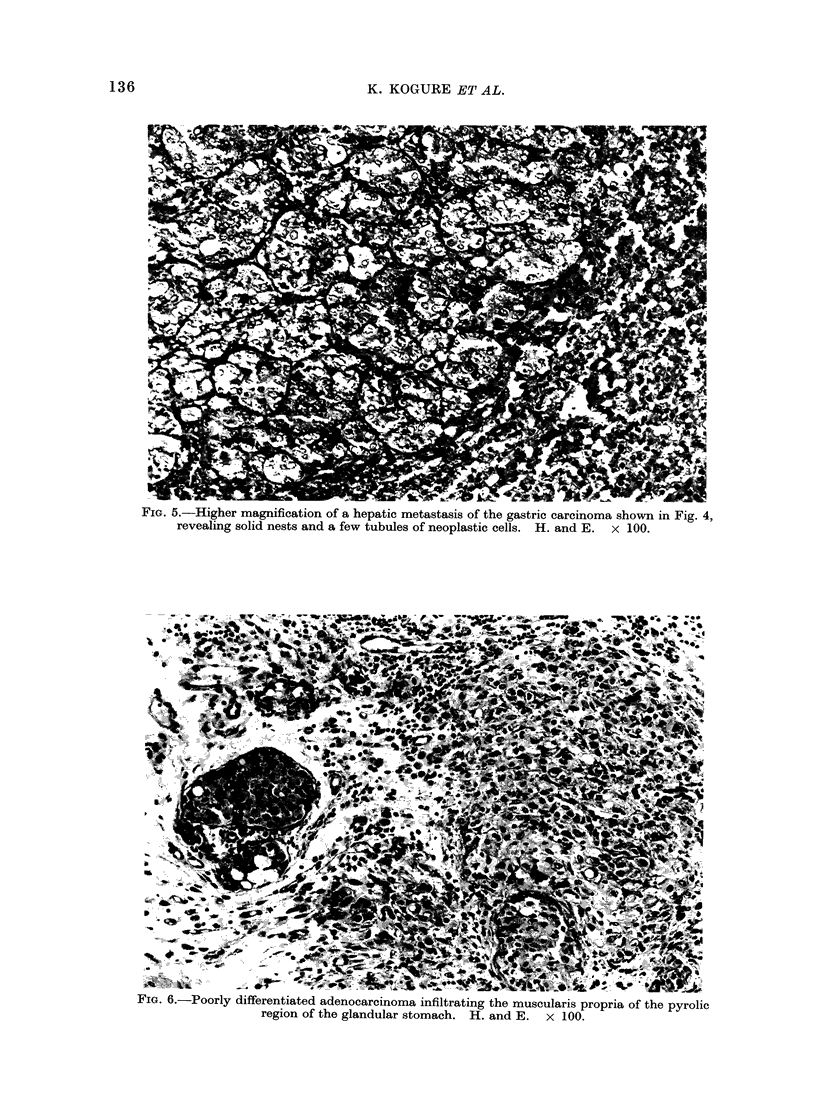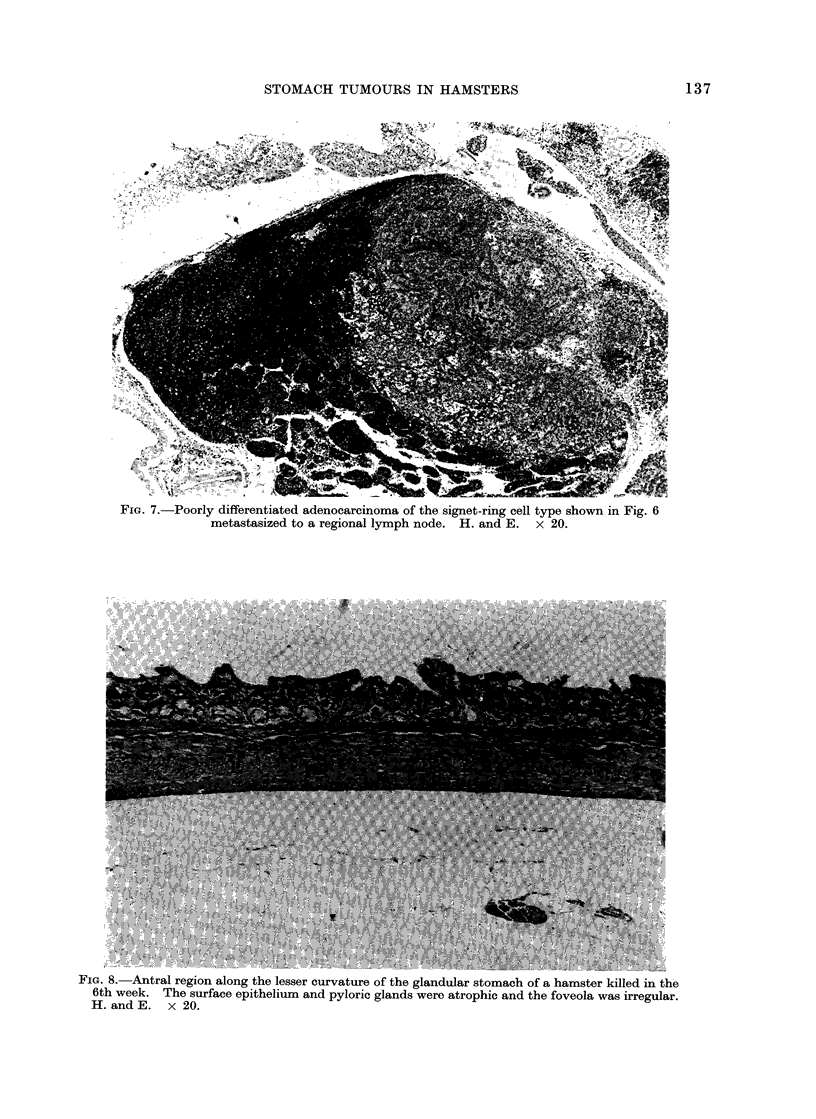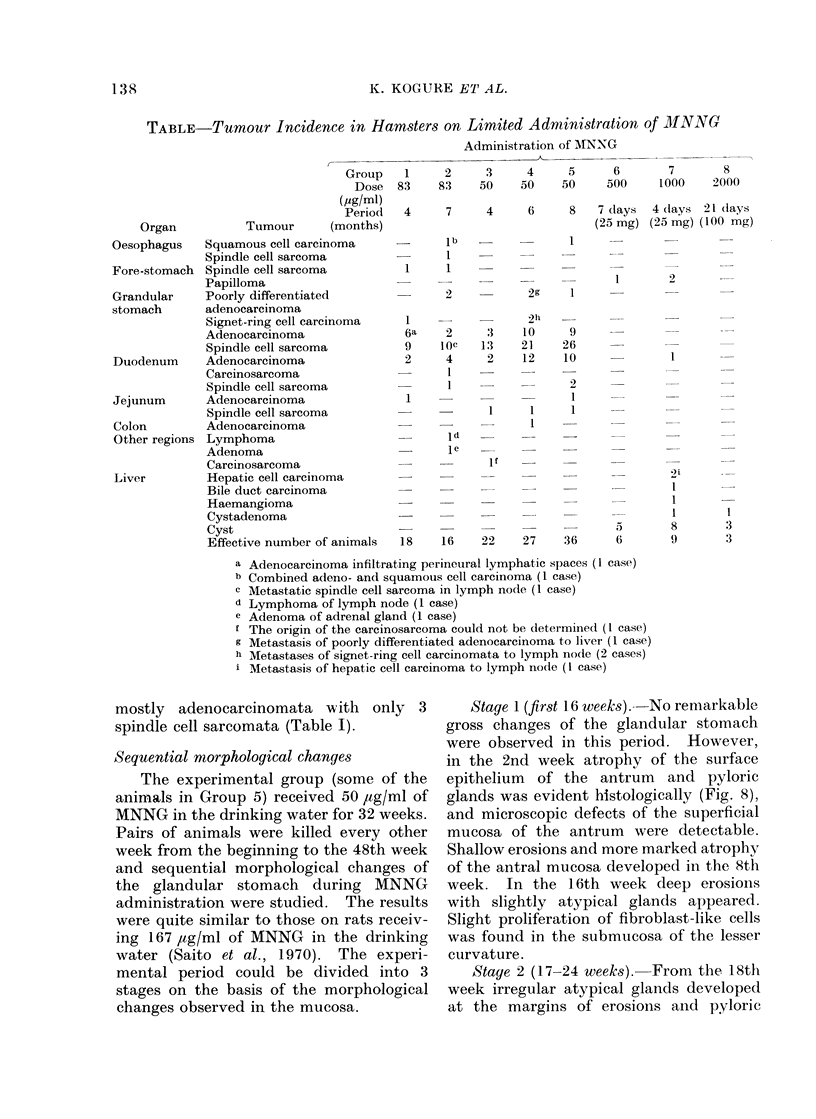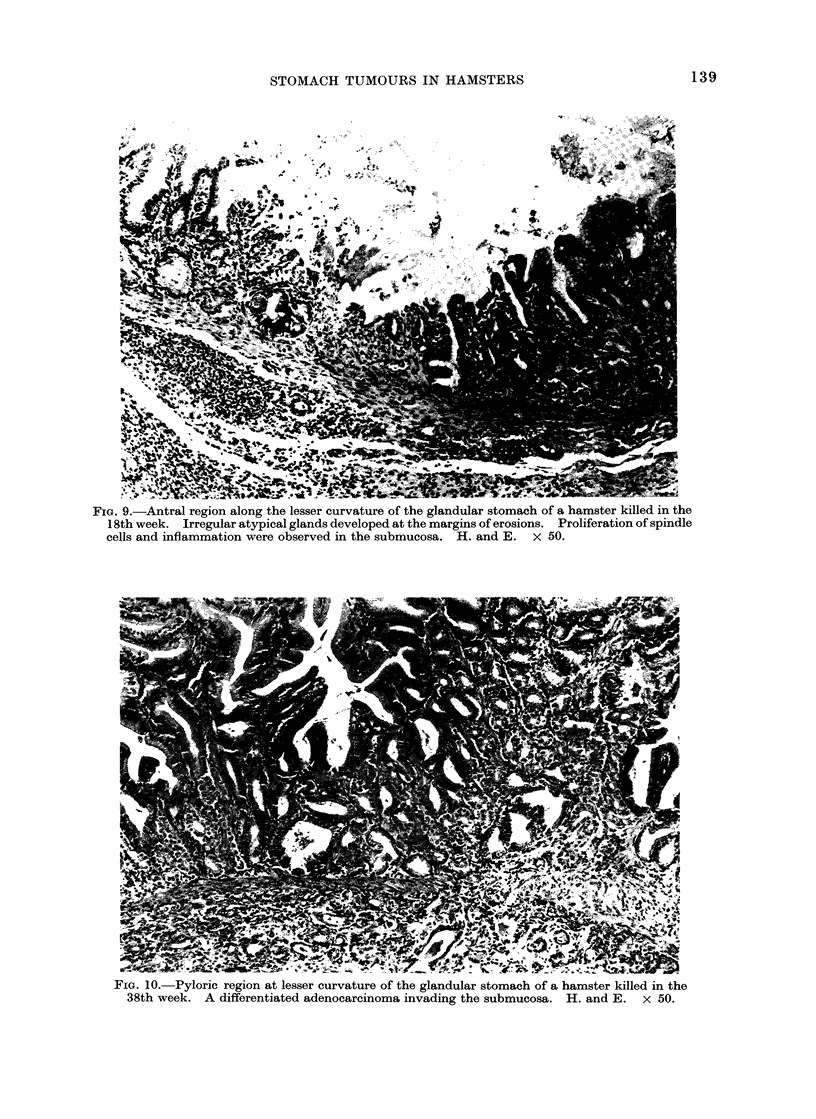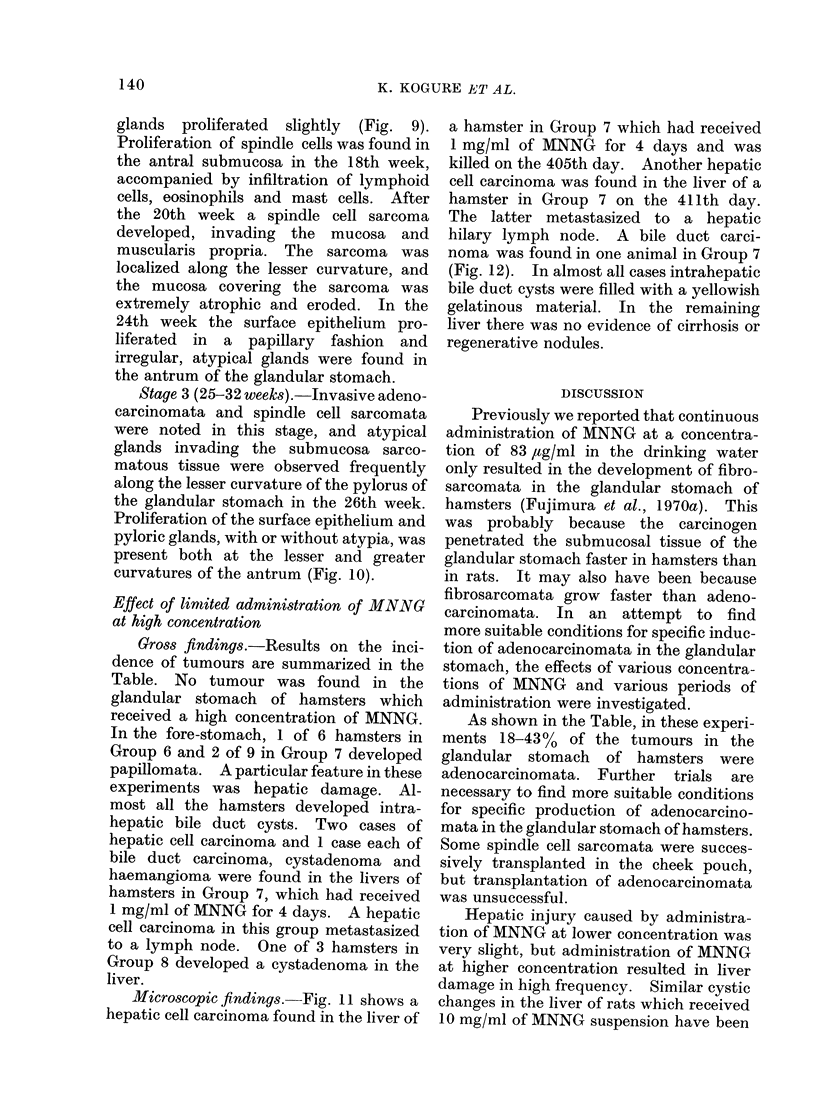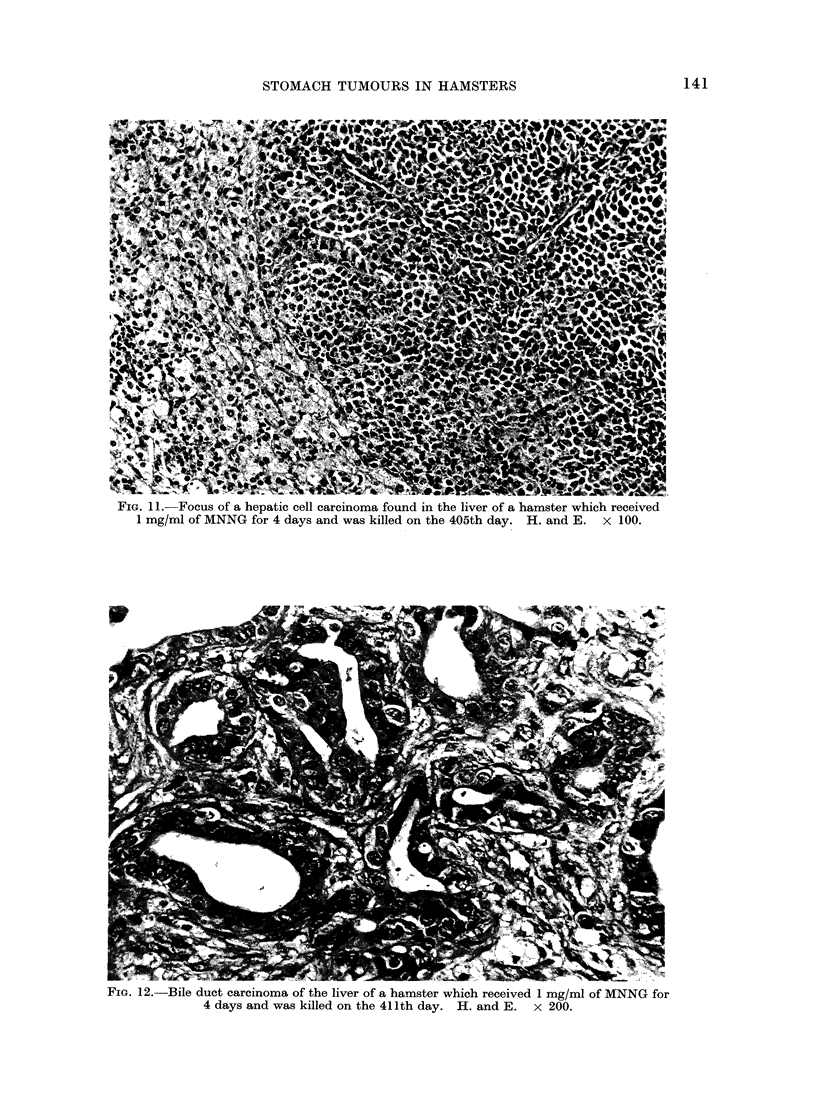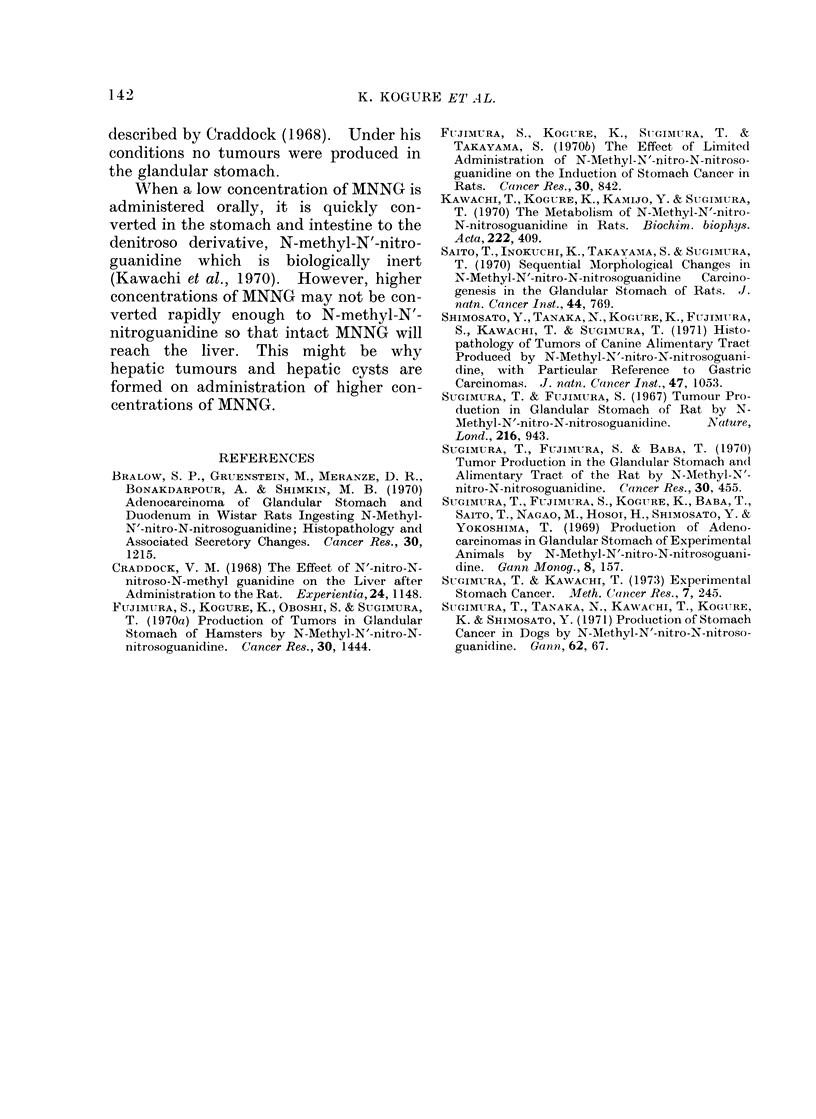# Further Studies on Induction of Stomach Cancer in Hamsters by N-methyl-N′-nitro-N-nitrosoguanidine*

**DOI:** 10.1038/bjc.1974.49

**Published:** 1974-02

**Authors:** K. Kogure, H. Sasadaira, T. Kawachi, Y. Shimosato, A. Tokunaga, S. Fujimura, T. Sugimura

## Abstract

**Images:**


*Br. J. Cancer* (1974) **29**, 132

### FURTHER STUDIES ON INDUCTION OF STOMACH CANCER IN HAMSTERS BY N-METHYL-N′-NITRO-N-NITROSOGUANIDINE*

K. KOGURE, H. SASADAIRA, T. KAWACHI, Y. SHIMOSATO, A. TOKUNAGA, S. FUJIMURA AND T. SUGIMURA

*From the Biochemistry Division and Pathology Division, National Cancer Center Research Institute, Tsukiji, Chuo-ku, Tokyo, Japan* 104

Received 30 August 1973. Accepted 20 October 1973

**Summary.—Oral administration of N-methyl-N′-nitro-N-nitrosoguanidine (MNNG) to hamsters at a concentration of 50–83 μg/ml in the drinking water resulted in a high incidence of tumours in the glandular stomach. Short-term administration of MNNG for 4–6 months resulted in more adenocarcinomata in the glandular stomach than long-term administration for 7–8 months. One case of metastasis of an adenocarcinoma of the glandular stomach to the liver and 2 cases of metastasis to the regional lymph nodes were found. Spindle cell sarcomata in the glandular stomach and adenocarcinomata in the duodenum were also often produced.**

**Oral administration of MNNG at the very high concentration of 500–2000 μg/ml induced a hepatic cell carcinoma, intrahepatic bile duct carcinomata, bile duct cystadenomata and cystic dilatation, and a haemangioma in the liver but no tumour in the glandular stomach.**

**Sequential morphological studies on the glandular stomach of hamsters receiving 50 μg/ml of MNNG in the drinking water showed 3 stages of change of the mucosa. The mucosa became atrophic and eroded in the first 16 weeks. Irregular atypical glands developed at the margins of erosions and proliferation of spindle cells in the submucosa were found after 18 weeks. Spindle cell sarcomata developed in animals after 20 weeks. Adenocarcinomata developed between 25 and 32 weeks.**

A SIMPLE and reliable method of inducing adenocarcinomata in the glandular stomach of rats (Bralow *et al.*, 1970; Fujimura *et al.*, 1970*b*; Sugimura and Fujimura, 1967; Sugimura, Fujimura and Baba, 1970) and the stomach of dogs (Shimosato *et al.*, 1971; Sugimura *et al.*, 1971) with MNNG was established and reviewed recently (Sugimura and Kawachi, 1973). Although production of tumours in the glandular stomach of hamsters by continuous administration of MNNG has been reported previously (Fujimura *et al.*, 1970*a*), most of the tumours observed were fibrosarcomata. In an attempt to establish more suitable conditions for production of adenocarcinomata in the glandular stomach of hamsters, the effect of a limited period of administration of MNNG solution of lower concentration was examined.

### MATERIALS AND METHODS

*Animals.*—Male Golden hamsters were purchased from CLEA Co., Tokyo. They were 6 weeks old and weighed about 60 g at the beginning of experiments. They were housed 2 to a cage and maintained on a commercial diet (CE-2). Experimental animals were divided into 8 groups of 10–40 animals. Groups 1 and 2 received 83 μg/ml of MNNG in the drinking water for 4 months and 7 months respectively. The animals in these 2 groups were autopsied when they died or become moribund. Groups 3, 4 and 5 received 50 μg/ml of MNNG in the drinking water

* This is Paper XII in a series dealing with the production of gastric cancer. This work was supported by Grants from the Ministry of Education, the Ministry of Health and Welfare and the Society for Promotion of Cancer Research.

for 4, 6 and 8 months respectively. Pairs of animals in Group 5 were killed every other week from the first to the 48th week of the experiment to study sequential morphological changes of the mucosa of the glandular stomach. After the 48th week animals were autopsied when they became moribund. Group 6 received 500 $\mu$g/ml of MNNG in the drinking water for 7 days and Group 7 received 1 mg/ml of MNNG in the drinking water for only 4 days. The total intake of MNNG by each hamster was 25 mg in these groups, calculated from the amount of drinking water consumed; spillage was allowed for in calculating the dose. Hamsters in Group 8 received 2 mg/ml of MNNG in the drinking water for 21 days and their total intake of carcinogen was 100 mg. All animals which were killed or died during the experimental period were examined histologically.

*Chemicals.*—MNNG was purchased from Aldrich Chemical Co. Inc., Milwaukee, Wis., U.S.A. or K and K Laboratories, Plainview, N.Y., U.S.A. MNNG was dissolved in deionized water at a concentration of 1 mg/ml and was stored in a dark bottle in a cold place. A solution of MNNG in deionized water at 1 mg/ml is fairly stable when stored in dark and cold (Sugimura *et al.*, 1969). The stock solution of MNNG was diluted with tap water to the required concentration. Diluted solution was prepared every other day. Hamsters were allowed to drink *ad libitum.*

### RESULTS

### *Effect of limited administration of MNNG at low concentration*

*Gross findings.*—Results on the incidence of tumours are summarized in the Table. All tumours were found in the upper alimentary tract except for one tumour each in an adrenal gland, the colon and a lymph node. The first animals with tumour were observed on Days 177, 211, 168, 182 and 126 in Groups 1, 2, 3, 4 and 5 respectively. As summarized in the Table, 36 cases of carcinoma and 79 cases of spindle cell sarcoma were found among 117 cases of tumour of the glandular stomach in these experiments. The percentages of adenocarcinomata among the total tumours produced in the glandular stomach of Groups 1, 2, 3, 4 and 5 were 43, 28, 18, 38 and 25% respectively. At a concentration of 83 $\mu$g/ml administration for 4 months induced more adenocarcinomata than administration for 7 months. At a concentration of 50 $\mu$g/ml administration for 6 months resulted in more adenocarcinomata than administration for 4 or 8 months. A squamous cell carcinoma and a spindle cell sarcoma were found in the oesophagus of hamsters in Group 2. Two cases of spindle cell sarcoma were found in the fore-stomach of animals in Groups 1 and 2. Almost all the tumours of the glandular stomach were localized along the lesser curvature of the pyloric region of the antrum (Fig. 1). However, among these tumours, spindle cell sarcomata were observed more frequently than adenocarcinomata, irrespective of the conditions of MNNG administration. All the tumours found in the duodenum were proximal to the papilla vateri. All tumours in the jejunum were localized in the upper part.

*Microscopic findings.*—Fig. 2 shows a signet-ring cell carcinoma found in the glandular stomach of a hamster in Group 1 which had received MNNG for 4 months and was killed 4 months later. A signet-ring cell carcinoma was found in one animal in Group 1 and 2 in Group 4. Two animals in Group 2, 2 in Group 4 and 1 in Group 5 developed a poorly differentiated adenocarcinoma. All the other adenocarcinomata were well differentiated but invaded the submucosal layer, often with partial serosal involvement (Fig. 3). Invasion of differentiated adenocarcinoma cells into the perineural lymphatics of the glandular stomach was found in one animal in Group 1. A poorly differentiated type of gastric adenocarcinoma in an animal in Group 4 produced a metastasis in the liver (Fig. 4 and 5) and 2 other cases of poorly differentiated adenocarcinomata in Group 4 metastasized to the regional lymph nodes where signet-ring type tumour cells were also found (Fig. 6 and 7). A metastasis of a spindle cell sarcoma to a regional lymph node was found in one animal in Group 2. The 34 tumours in the duodenum were

Fig. 1.—Gross appearance of cancer in the glandular stomach of a hamster. An adenocarcinoma invaded the antrum of the glandular stomach of a hamster. The animal was killed 4 months after administration of MNNG for 4 months.

Fig. 2.—Neoplastic signet-ring cells infiltrating the muscularis propria of the pyloric region of the glandular stomach. H. and E. × 100.

FIG. 3.—Differentiated tubular adenocarcinoma invading the submucosa of the glandular stomach. H. and E. × 50.

FIG. 4.—A poorly differentiated gastric tumour composed of solid nests and a few tubules. The tumour was ulcerated and reached the serosa. H. and E. × 20.

FIG. 5.—Higher magnification of a hepatic metastasis of the gastric carcinoma shown in Fig. 4, revealing solid nests and a few tubules of neoplastic cells. H. and E. × 100.

FIG. 6.—Poorly differentiated adenocarcinoma infiltrating the muscularis propria of the pyrolic region of the glandular stomach. H. and E. × 100.

Fig. 7.—Poorly differentiated adenocarcinoma of the signet-ring cell type shown in Fig. 6 metastasized to a regional lymph node. H. and E. × 20.

Fig. 8.—Antral region along the lesser curvature of the glandular stomach of a hamster killed in the 6th week. The surface epithelium and pyloric glands were atrophic and the foveola was irregular. H. and E. × 20.

TABLE—*Tumour Incidence in Hamsters on Limited Administration of MNNG*

| Organ | Tumour | Administration of MNNG: Group 1 | 2 | 3 | 4 | 5 | 6 | 7 | 8 |
|---|---|---|---|---|---|---|---|---|---|
| | Dose (μg/ml) | 83 | 83 | 50 | 50 | 50 | 500 | 1000 | 2000 |
| | Period (months) | 4 | 7 | 4 | 6 | 8 | 7 days (25 mg) | 4 days (25 mg) | 21 days (100 mg) |
| Oesophagus | Squamous cell carcinoma | — | 1[b] | — | — | 1 | — | — | — |
| | Spindle cell sarcoma | — | 1 | — | — | — | — | — | — |
| Fore-stomach | Spindle cell sarcoma | 1 | 1 | — | — | — | — | — | — |
| | Papilloma | — | — | — | — | — | 1 | 2 | — |
| Grandular stomach | Poorly differentiated adenocarcinoma | — | 2 | — | 2[g] | 1 | — | — | — |
| | Signet-ring cell carcinoma | 1 | — | — | 2[h] | — | — | — | — |
| | Adenocarcinoma | 6[a] | 2 | 3 | 10 | 9 | — | — | — |
| | Spindle cell sarcoma | 9 | 10[c] | 13 | 21 | 26 | — | — | — |
| Duodenum | Adenocarcinoma | 2 | 4 | 2 | 12 | 10 | — | 1 | — |
| | Carcinosarcoma | — | 1 | — | — | — | — | — | — |
| | Spindle cell sarcoma | — | 1 | — | — | 2 | — | — | — |
| Jejunum | Adenocarcinoma | 1 | — | — | — | 1 | — | — | — |
| | Spindle cell sarcoma | — | — | 1 | 1 | 1 | — | — | — |
| Colon | Adenocarcinoma | — | — | — | 1 | — | — | — | — |
| Other regions | Lymphoma | — | 1[d] | — | — | — | — | — | — |
| | Adenoma | — | 1[e] | — | — | — | — | — | — |
| | Carcinosarcoma | — | — | 1[f] | — | — | — | — | — |
| Liver | Hepatic cell carcinoma | — | — | — | — | — | — | 2[i] | — |
| | Bile duct carcinoma | — | — | — | — | — | — | 1 | — |
| | Haemangioma | — | — | — | — | — | — | 1 | — |
| | Cystadenoma | — | — | — | — | — | — | 1 | 1 |
| | Cyst | — | — | — | — | — | 5 | 8 | 3 |
| | Effective number of animals | 18 | 16 | 22 | 27 | 36 | 6 | 9 | 3 |

[a] Adenocarcinoma infiltrating perineural lymphatic spaces (1 case)
[b] Combined adeno- and squamous cell carcinoma (1 case)
[c] Metastatic spindle cell sarcoma in lymph node (1 case)
[d] Lymphoma of lymph node (1 case)
[e] Adenoma of adrenal gland (1 case)
[f] The origin of the carcinosarcoma could not be determined (1 case)
[g] Metastasis of poorly differentiated adenocarcinoma to liver (1 case)
[h] Metastases of signet-ring cell carcinomata to lymph node (2 cases)
[i] Metastasis of hepatic cell carcinoma to lymph node (1 case)

mostly adenocarcinomata with only 3 spindle cell sarcomata (Table I).

*Sequential morphological changes*

The experimental group (some of the animals in Group 5) received 50 μg/ml of MNNG in the drinking water for 32 weeks. Pairs of animals were killed every other week from the beginning to the 48th week and sequential morphological changes of the glandular stomach during MNNG administration were studied. The results were quite similar to those on rats receiving 167 μg/ml of MNNG in the drinking water (Saito *et al.*, 1970). The experimental period could be divided into 3 stages on the basis of the morphological changes observed in the mucosa.

*Stage* 1 (*first* 16 *weeks*).—No remarkable gross changes of the glandular stomach were observed in this period. However, in the 2nd week atrophy of the surface epithelium of the antrum and pyloric glands was evident histologically (Fig. 8), and microscopic defects of the superficial mucosa of the antrum were detectable. Shallow erosions and more marked atrophy of the antral mucosa developed in the 8th week. In the 16th week deep erosions with slightly atypical glands appeared. Slight proliferation of fibroblast-like cells was found in the submucosa of the lesser curvature.

*Stage* 2 (17–24 *weeks*).—From the 18th week irregular atypical glands developed at the margins of erosions and pyloric

FIG. 9.—Antral region along the lesser curvature of the glandular stomach of a hamster killed in the 18th week. Irregular atypical glands developed at the margins of erosions. Proliferation of spindle cells and inflammation were observed in the submucosa. H. and E. × 50.

FIG. 10.—Pyloric region at lesser curvature of the glandular stomach of a hamster killed in the 38th week. A differentiated adenocarcinoma invading the submucosa. H. and E. × 50.

glands proliferated slightly (Fig. 9). Proliferation of spindle cells was found in the antral submucosa in the 18th week, accompanied by infiltration of lymphoid cells, eosinophils and mast cells. After the 20th week a spindle cell sarcoma developed, invading the mucosa and muscularis propria. The sarcoma was localized along the lesser curvature, and the mucosa covering the sarcoma was extremely atrophic and eroded. In the 24th week the surface epithelium proliferated in a papillary fashion and irregular, atypical glands were found in the antrum of the glandular stomach.

*Stage* 3 (25–32 *weeks*).—Invasive adenocarcinomata and spindle cell sarcomata were noted in this stage, and atypical glands invading the submucosa sarcomatous tissue were observed frequently along the lesser curvature of the pylorus of the glandular stomach in the 26th week. Proliferation of the surface epithelium and pyloric glands, with or without atypia, was present both at the lesser and greater curvatures of the antrum (Fig. 10).

*Effect of limited administration of MNNG at high concentration*

*Gross findings.*—Results on the incidence of tumours are summarized in the Table. No tumour was found in the glandular stomach of hamsters which received a high concentration of MNNG. In the fore-stomach, 1 of 6 hamsters in Group 6 and 2 of 9 in Group 7 developed papillomata. A particular feature in these experiments was hepatic damage. Almost all the hamsters developed intrahepatic bile duct cysts. Two cases of hepatic cell carcinoma and 1 case each of bile duct carcinoma, cystadenoma and haemangioma were found in the livers of hamsters in Group 7, which had received 1 mg/ml of MNNG for 4 days. A hepatic cell carcinoma in this group metastasized to a lymph node. One of 3 hamsters in Group 8 developed a cystadenoma in the liver.

*Microscopic findings.*—Fig. 11 shows a hepatic cell carcinoma found in the liver of a hamster in Group 7 which had received 1 mg/ml of MNNG for 4 days and was killed on the 405th day. Another hepatic cell carcinoma was found in the liver of a hamster in Group 7 on the 411th day. The latter metastasized to a hepatic hilary lymph node. A bile duct carcinoma was found in one animal in Group 7 (Fig. 12). In almost all cases intrahepatic bile duct cysts were filled with a yellowish gelatinous material. In the remaining liver there was no evidence of cirrhosis or regenerative nodules.

### DISCUSSION

Previously we reported that continuous administration of MNNG at a concentration of 83 $\mu$g/ml in the drinking water only resulted in the development of fibrosarcomata in the glandular stomach of hamsters (Fujimura *et al.*, 1970*a*). This was probably because the carcinogen penetrated the submucosal tissue of the glandular stomach faster in hamsters than in rats. It may also have been because fibrosarcomata grow faster than adenocarcinomata. In an attempt to find more suitable conditions for specific induction of adenocarcinomata in the glandular stomach, the effects of various concentrations of MNNG and various periods of administration were investigated.

As shown in the Table, in these experiments 18–43% of the tumours in the glandular stomach of hamsters were adenocarcinomata. Further trials are necessary to find more suitable conditions for specific production of adenocarcinomata in the glandular stomach of hamsters. Some spindle cell sarcomata were successively transplanted in the cheek pouch, but transplantation of adenocarcinomata was unsuccessful.

Hepatic injury caused by administration of MNNG at lower concentration was very slight, but administration of MNNG at higher concentration resulted in liver damage in high frequency. Similar cystic changes in the liver of rats which received 10 mg/ml of MNNG suspension have been

FIG. 11.—Focus of a hepatic cell carcinoma found in the liver of a hamster which received 1 mg/ml of MNNG for 4 days and was killed on the 405th day. H. and E. × 100.

FIG. 12.—Bile duct carcinoma of the liver of a hamster which received 1 mg/ml of MNNG for 4 days and was killed on the 411th day. H. and E. × 200.

described by Craddock (1968). Under his conditions no tumours were produced in the glandular stomach.

When a low concentration of MNNG is administered orally, it is quickly converted in the stomach and intestine to the denitroso derivative, N-methyl-N′-nitroguanidine which is biologically inert (Kawachi *et al.*, 1970). However, higher concentrations of MNNG may not be converted rapidly enough to N-methyl-N′-nitroguanidine so that intact MNNG will reach the liver. This might be why hepatic tumours and hepatic cysts are formed on administration of higher concentrations of MNNG.

### REFERENCES

BRALOW, S. P., GRUENSTEIN, M., MERANZE, D. R., BONAKDARPOUR, A. & SHIMKIN, M. B. (1970) Adenocarcinoma of Glandular Stomach and Duodenum in Wistar Rats Ingesting N-Methyl-N′-nitro-N-nitrosoguanidine; Histopathology and Associated Secretory Changes. *Cancer Res.*, **30**, 1215.

CRADDOCK, V. M. (1968) The Effect of N′-nitro-N-nitroso-N-methyl guanidine on the Liver after Administration to the Rat. *Experientia*, **24**, 1148.

FUJIMURA, S., KOGURE, K., OBOSHI, S. & SUGIMURA, T. (1970*a*) Production of Tumors in Glandular Stomach of Hamsters by N-Methyl-N′-nitro-N-nitrosoguanidine. *Cancer Res.*, **30**, 1444.

FUJIMURA, S., KOGURE, K., SUGIMURA, T. & TAKAYAMA, S. (1970*b*) The Effect of Limited Administration of N-Methyl-N′-nitro-N-nitrosoguanidine on the Induction of Stomach Cancer in Rats. *Cancer Res.*, **30**, 842.

KAWACHI, T., KOGURE, K., KAMIJO, Y. & SUGIMURA, T. (1970) The Metabolism of N-Methyl-N′-nitro-N-nitrosoguanidine in Rats. *Biochim. biophys. Acta*, **222**, 409.

SAITO, T., INOKUCHI, K., TAKAYAMA, S. & SUGIMURA, T. (1970) Sequential Morphological Changes in N-Methyl-N′-nitro-N-nitrosoguanidine Carcinogenesis in the Glandular Stomach of Rats. *J. natn. Cancer Inst.*, **44**, 769.

SHIMOSATO, Y., TANAKA, N., KOGURE, K., FUJIMURA, S., KAWACHI, T. & SUGIMURA, T. (1971) Histopathology of Tumors of Canine Alimentary Tract Produced by N-Methyl-N′-nitro-N-nitrosoguanidine, with Particular Reference to Gastric Carcinomas. *J. natn. Cancer Inst.*, **47**, 1053.

SUGIMURA, T. & FUJIMURA, S. (1967) Tumour Production in Glandular Stomach of Rat by N-Methyl-N′-nitro-N-nitrosoguanidine. *Nature, Lond.*, **216**, 943.

SUGIMURA, T., FUJIMURA, S. & BABA, T. (1970) Tumor Production in the Glandular Stomach and Alimentary Tract of the Rat by N-Methyl-N′-nitro-N-nitrosoguanidine. *Cancer Res.*, **30**, 455.

SUGIMURA, T., FUJIMURA, S., KOGURE, K., BABA, T., SAITO, T., NAGAO, M., HOSOI, H., SHIMOSATO, Y. & YOKOSHIMA, T. (1969) Production of Adenocarcinomas in Glandular Stomach of Experimental Animals by N-Methyl-N′-nitro-N-nitrosoguanidine. *Gann Monog.*, **8**, 157.

SUGIMURA, T. & KAWACHI, T. (1973) Experimental Stomach Cancer. *Meth. Cancer Res.*, **7**, 245.

SUGIMURA, T., TANAKA, N., KAWACHI, T., KOGURE, K. & SHIMOSATO, Y. (1971) Production of Stomach Cancer in Dogs by N-Methyl-N′-nitro-N-nitrosoguanidine. *Gann*, **62**, 67.